# Metabolomics analysis of patients with *Schistosoma japonicum* infection based on UPLC-MS method

**DOI:** 10.1186/s13071-024-06429-9

**Published:** 2024-08-20

**Authors:** Junhui Li, Jie Jiang, Yi Zhu, Yu Zhang, Jiang Zhu, Yingzi Ming

**Affiliations:** 1 Center for Organ Transplantation, Third Xiangya Hospital, Central South University, Changsha, 410013 Hunan China; 2 Key Laboratory of Translational Research in Transplantion Medicine of National Health Commission, Third Xiangya Hospital, Central South University, Changsha, 410013 Hunan China; 3 Hunan Province Clinical Research Center for Infectious Diseases, Changsha, 410013 Hunan China

**Keywords:** *Schistosoma japonicum*, UPLC-MS, Biomarker, Metabolomics

## Abstract

**Background:**

Schistosomiasis is still one of the most serious parasitic diseases. Evidence showed that the metabolite profile in serum can potentially act as a marker for parasitic disease diagnosis and evaluate disease progression and prognosis. However, the serum metabolome in patients with *Schistosoma japonicum* infection is not well defined. In this study, we investigated the metabolite profiles of patients with chronic and with advanced *S. japonicum* infection.

**Methods:**

The sera of 33 chronic *S. japonicum* patients, 15 patients with advanced schistosomiasis and 17 healthy volunteers were collected. Samples were extracted for metabolites and analyzed with ultra-performance liquid chromatography-mass spectrometry (UPLC-MS).

**Results:**

We observed significant differences in metabolite profiles in positive and negative ion modes between patients with advanced and chronic *S. japonicum* infection. In patients with chronic *S. japonicum* infection, 199 metabolites were significantly upregulated while 207 metabolites were downregulated in advanced infection. These differential metabolites were mainly concentrated in steroid hormone biosynthesis, cholesterol metabolism and bile secretion pathways. We also found that certain bile acid levels were significantly upregulated in the progression from chronic to advanced *S. japonicum* infection. In receiver operator characteristic (ROC) analysis, we identified three metabolites with area under the curve (AUC) > 0.8, including glycocholic (GCA), glycochenodeoxycholate (GCDCA) and taurochenodeoxycholic acid (TCDCA) concentrated in cholesterol metabolism, biliary secretion and primary bile acid biosynthesis.

**Conclusions:**

This study provides evidence that GCA, GCDCA and TCDCA can potentially act as novel metabolite biomarkers to distinguish patients in different stages of *S. japonicum* infection. This study will contribute to the understanding of the metabolite mechanisms of the transition from chronic to advanced *S. japonicum* infection, although more studies are needed to validate this potential role and explore the underlying mechanisms.

**Graphical Abstract:**

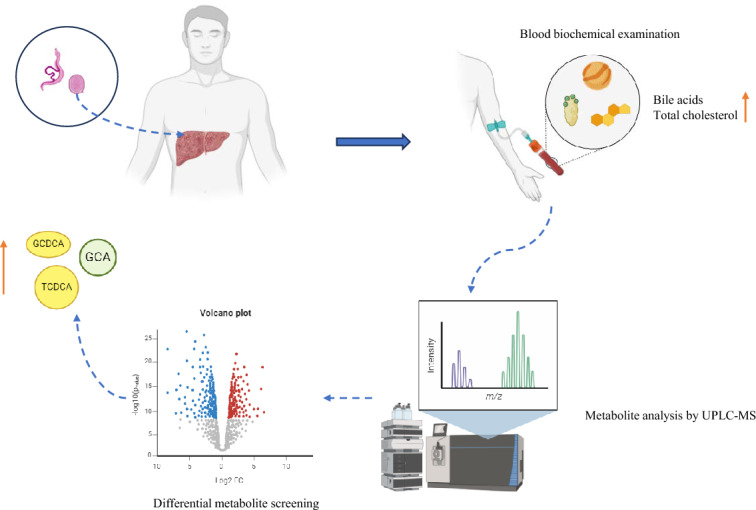

**Supplementary Information:**

The online version contains supplementary material available at 10.1186/s13071-024-06429-9.

## Background

Schistosomiasis is one of the most serious parasitic diseases, second only to malaria in the global list of neglected tropical diseases, and one of the most devastating tropical parasitic diseases [1]. It is estimated that 240 million people are currently infected worldwide, and > 700 million people in 76 countries and territories are affected, mainly in tropical and subtropical regions [2–6]. Schistosomiasis caused by *Schistosoma japonicum* is much more severe than the disease caused by other *Schistosoma* species [7]. After eggs have been deposited in a patient’s liver, intestine and spleen, they can persist for a long time, leading to chronic damage and liver fibrosis, a stage known as chronic *S. japonicum* infection. Patients diagnosed with chronic *S. japonicum* infection usually are slightly symptomatic or even asymptomatic. If patients have recurrent infections, even with treatment, after 5–10 years they will develop advanced *S. japonicum* infection, resulting in cirrhotic ascites, portal hypertension and a marked reduction in quality of life [8].

Currently, some advances have been achieved in diagnosing *S. japonicum* infection. Nonetheless, *S. japonicum* infection diagnosis relies on traditional methods such as the Kato-Katz (KK) technique, miracidial hatching method and antigen or antibody-based detection [9–11]. These conventional diagnostic strategies show low sensitivity and specificity and are closely related to sample collection [10]. Nucleic acid-based techniques, including PCR-based methods, cell-free DNA-based detection and CRISPR-Cas12/13-mediated detection, are characterized by high sensitivity and specificity but are limited because they require expensive equipment, reagents and experienced personnel [9, 12]. Thus, there is an urgent need to develop new diagnostic methods [13]. Recent studies showed significant changes in serum metabolites in different stages of *S. japonicum* infection in mice, and serum metabolites have potential to act as an indicator for disease burden and assist early diagnosis [14, 15]. However, the serum metabolome has not been explored in patients infected with *S. japonicum*. Animal studies indicated that further studies on metabolite responses in patients with advanced and chronic *S. japonicum* infection may help us better understand the mechanisms of disease progression and shed light on tools for early diagnosis of *S. japonicum* infection.

Metabolomics involve the study of multivariate metabolite responses between the host internal environment and complex parasite infection, which can reflect host regulation of parasite growth and development as well as disease progression [16]. In this study, ultra-performance liquid chromatography-mass spectrometry (UPLC-MS)-based metabolomic method was applied to analyze serum metabolites changes in *S. japonicum* infection. Alterations in serum metabolites in different stages of *S. japonicum* infection were observed. This study provided evidence that the altered metabolites seem to have potential to be novel metabolite biomarkers to distinguish patients in different stages of *S. japonicum* infection. Also, this study will contribute to the understanding of the metabolite mechanisms of the transition from chronic to advanced *S. japonicum* infection.

## Methods

### Sample collection and clinical information

Patients with *S. japonicum* infection are diagnosed according to the National Standardized Diagnostic Criteria for *S. japonicum* infection (WS261-2006) of the Ministry of Health of China [17]. The diagnostic criteria for chronic schistosomiasis japonica were as follows: (i) a history of exposure to endemic water in endemic areas of *S. japonicum*; (ii) medical history of diagnosis of schistosomiasis based on Kato-Katz or indirect hemagglutination test and received praziquantel treatment; (iii) asymptomatic or intermittent abdominal pain, diarrhea or purulent and bloody stool; (iv) positive ovum test in the patient stool or positive serum immunological test; (v) B-ultrasound examination indicated fibrosis, without ascites, portal hypertension and splenomegaly, etc. [18]. Patients with advanced schistosomiasis should meet the following conditions: (i) history of repeated or prolonged exposure to water in endemic areas; (ii) history of treatment for *S. japonicum* infection; (iii) positive ovum stool test or positive serum immunological test; (iv) liver fibrosis; (v) one or more complications of splenomegaly, hypersplenism, portal hypertension or ascites. In this study, all patients (≥ 18 years old) with *S. japonicum* infection who visited the Xiangyue Hospital of Hunan Province from January 2022 to August 2022 and agreed to participate in this study were included. Patients with the following diseases were excluded from research: hybrid liver diseases; combined infection with other viruses, such as HAV, HBV, HCV or HDV; primary liver cancer or other malignant tumors; medium and severe fatty liver; severe liver dysfunction. Healthy volunteers were from the same area as those who received health examinations in the same hospital and agreed to participate in this study. According to the inclusion and exclusion criteria, 33 patients with chronic *S. japonicum* infection and 15 patients with advanced *S. japonicum* infection and 17 healthy volunteers were included. The serum and clinical information on the patients with *S. japonicum* infection in the hospital was collected. The related clinical information is shown in Table 1. Blood (5 ml) was collected early in the morning from all participants who fasted for 10 h and was coagulated at room temperature. Serum was obtained by centrifugation at 3000 rpm for 5 min and stored at − 80 °C. We used the same standard for all samples. This study was approved by the Institutional Review Board (IRB) of the Third Xiangya Hospital of Central South University (21149), and written informed consent was obtained from all participants.

**Table 1:** Clinical characteristics of participants

	CSJ (n = 33)	ASJ (n = 15)	CON (n = 17)	P value
Female, (n%)	12 (36.4%)	8 (53.3%)	7 (35.3%)	0.484^a^
Male, (n%)	21 (63.6%)	7 (46.7%)	10 (64.7%)	
Age (years), mean ± SD	58.27 ± 10.84	64.47 ± 10.60	57.82 ± 8.58	0.122^b^
TBA (umol/l), mean ± SD	3.99 ± 2.32	13.04 ± 8.30	2.51 ± 1.47	< 0.001^b^
GLU (mmol/l), mean ± SD	5.79 ± 1.10	6.66 ± 3.46	5.11 ± 0.33	0.066^b^
TG (mmol/l), mean ± SD	1.87 ± 1.54	1.29 ± 0.80	1.21 ± 0.40	0.126^b^
TC (mmol/l), mean ± SD	5.33 ± 1.08	5.10 ± 1.14	4.66 ± 0.82	0.025^b^
HDL-C (mmol/l), mean ± SD	1.54 ± 0.38	1.61 ± 0.42	1.40 ± 0.28	0.057^b^
LDL-C (mmol/l), mean ± SD	3.50 ± 1.02	3.21 ± 0.96	2.71 ± 0.61	0.005^b^

^a^Fisher’s exact test

^b^ANOVA test

*CSJ* Chronic *Schistosoma japonicum* infection group, *ASJ* advanced *S. japonicum* infection group, *CON* control group, *GLU* glucose, *TG* triglyceride, *TBA* total bile acid, *TC* total cholesterol, *HDL-C* high-density lipoprotein cholesterol, *LDL-C* low-density lipoprotein cholesterol

### Serum sample analysis using UPLC-MS

#### Chemicals and reagents

Methanol (A454-4) and acetonitrile (A998-4) were all analytical grade (Thermo Fisher Scientific, USA); ammonia formate (17843-250G, Honeywell Fluka, USA) and formic acid (50144-50 ml, DIMKA, USA) were used. Water was supplied by ultra-pure water.

#### Sample preparation

Serum samples were stored at − 80 °C until analyzed. Metabolites were isolated from serum using methanol as described previously [19]; 700 µl extractant containing internal standard 1 (methanol: acetonitrile: water = 4:2:1, *V*/*V*/*V*) was added to 100 µl serum including quality control (QC) sample. After vortexing for 1 min, the samples were incubated at − 20 ℃ for 2 h and then centrifuged for 15 min at 25,000 g, 4 ℃; 600 µl of the supernatant was collected and dried. Then, 180 µl methanol:pure water (1:1 *v*/*v*) was added to resuspend that sample. After vortexing for 10 min, it was then centrifuged for 15 min at 25,000 g, 4 ℃. The supernatant was collected for LC-MS/MS analysis.

#### UPLC-MS analysis

This experiment used Waters UPLC I-Class Plus (Waters, USA) tandom Q Exactive high-resolution mass spectrometer (Thermo Fisher Scientific, USA) for separation and detection of metabolites.

Chromatographic separation was performed on a Waters ACQUITY UPLC BEH C18 column (1.7 μm, 2.1 × 100 mm, Waters, USA), and the column temperature was maintained at 45 °C. The mobile phase consisted of 0.1% formic acid (A) and acetonitrile (B) in positive mode, and in negative mode, the mobile phase consisted of 10 mM ammonium formate (A) and acetonitrile (B). The gradient conditions were as follows: 0–1 min, 2% B; 1–9 min, 2–98% B; 9–12 min, 98% B; 12–12.1 min, 98% B to 2% B; 12.1–15 min, 2% B. The flow rate was 0.35 ml/min, and the injection volume was 5 μl.

Q Exactive (Thermo Fisher Scientific, USA) was used to perform primary and secondary mass spectrometry data acquisition. The full scan range was 70–1050 m/z with a resolution of 70000, and the automatic gain control (AGC) target for MS acquisitions was set to 3e6 with a maximum ion injection time of 100 ms. The top three precursors were selected for subsequent MSMS fragmentation with a maximum ion injection time of 50 ms and resolution of 17,500; the AGC was 1e5. The stepped normalized collision energy was set to 20, 40 and 60 eV. ESI parameters were: sheath gas flow rate of 40, auxiliary gas flow rate of 10, positive ion mode spray voltage (|KV|) of 3.80, negative ion mode spray voltage (|KV|) of 3.20, capillary temperature of 320 °C and auxiliary gas heater temperature of 350 °C.

#### Metabolite ion peak extraction and metabolite identification

After quality control and pre-processing, ion peaks were extracted. The off-line data of mass spectrometry were imported into Compound Discoverer 3.3 (Thermo Fisher Scientific, USA) software, and metabolites were identified by comparison with standard references in the Beijing Genomics Institute metabolome database (BMBD), mzcloud database and chemspider online database. Then, the data were further analyzed and processed.

#### Data preprocessing

The result files from Compound Discoverer were input into MetaXto MetaX. Data preprocessing included: (i) data normalization to obtain relative peak areas by probabilistic quotient normalization (PQN) [20]; (ii) quality control-based robust loess signal correction to correct batch effect [19]; (iii) removal of metabolites with a coefficient of variation > 30% on the relative peak area in QC samples.

#### Quality control

Principal component analysis (PCA) was used to observe the overall distribution of QC for each group of samples and stability of the entire analysis process. PCA is an unsupervised pattern recognition method for statistical analysis of multidimensional data. Through orthogonal transformation, a group of variables that may be correlated is converted into a group of linear unrelated variables, which are called principal components after the transformation. This method is used to study how a few principal components can reveal the internal structure between multiple variables while keeping the original variable information. Log transformation and Pareto scaling were mainly used to compute PCA. The PCA plot reflects the real distribution of samples and was mainly used to observe the separation trend between sample groups and whether there were abnormal samples and to reflect the variability between and within groups from the original data. For QC samples, the better the QC samples aggregate, the more stable the instrument is and the better the reproducibility of the collected data.

#### Overall analysis

The identified metabolites were classified and annotated using Human Metabolome Database (HMBD). The HMBD contains chemical, molecular biological/biochemical and clinical information on metabolites and supports metabolic pathway and spectral searches. The function annotation of pathway was performed using Kyoto Encyclopedia of Genes and Genomes (KEGG) through the KEGG PATHWAY database (core of KEGG database) to determine the main biochemical metabolic pathways involved in the metabolites. Numerous metabolic pathways and the relationships between them can be found there. To investigate potential metabolite pathways involved in the progression of *S. japonicum* infection, we conducted KEGG pathway analysis with the differently expressed metabolites in different stages of *S. japonicum* infection. The differential abundance (DA) score was calculated according a previous publication [21]. DA = (no. of increased metabolites − no. of decreased metabolites)/no. of measured metabolites in a given pathway. The DA score can indicate the average and gross alterations of all measured metabolites in a pathway. A DA score of 1 indicates all metabolites in a pathway increased in abundance, while a DA score of − 1 denotes all metabolites decreased.

#### Screening the differences between groups

Partial least squares discriminant analysis (PLS-DA) and orthogonal partial least squares discriminant analysis (OPLS-DA) performed on preprocessed data in the R programming language version 4.0.3 (R Foundation for Statistical Computing, 2020) were used to evaluate the metabolite alterations between advanced and chronic *S. japonicum* infection. Unlike PCA, PLS-DA is a supervised statistical method. It can reflect the differences between classification groups better. This method uses partial least squares regression to establish a model between metabolite expression and sample categories to predict sample categories. After log 2 transformation of the data, a PLS-DA model was established between the comparative analysis groups (two groups of samples), the scaling method was Pareto, and a sevenfold cross validation was used for validation when building the model.

OPLS-DA is a combination of OSC and PLS-DA; it is an extension of PLS-DA, which can decompose X matrix information into two types of information related to Y and unrelated to Y, remove information irrelevant to classification and effectively reduce the complexity of the model without reducing the predictive ability of the model; this enhances the explanatory power of the model. Two hundred iterations of response permutation tests were performed to validate the OPLS-DA model and prevent model overfitting. Also, variable importance in projection (VIP) was used to measure the impact strength and explanatory power of each metabolite expression pattern on the classification and discrimination of each group of samples, which helped to screen the metabolic biomarkers. Generally, a > 1 VIP value could indicate metabolites that had a significant effect on distinct sample categories. VIP values, indicating the importance of variables for group discrimination, were obtained from the OPLS-DA model. Fold change analysis (FC analysis) and t-test were performed on the data. Fold change (FC) was obtained by FC analysis; *P*-value was obtained by t-test. *P*-value was used to assess statistical significance between two analyzed groups. Metabolites with VIP ≥ 1, fold change ≥ 1.2 or ≤ 0.83 and *P*-value < 0.05 were considered differential metabolites.

#### ROC curve and statistical analyses

ROC curve analysis was performed using SPSS software version 25.0 (SPSS Inc., Chicago, IL) to identify potential candidate biomarkers (area under the curve > 0.8) in the discovery set and to assess the predictive power of markers in metabolomics studies [22]. Pathway analyses of all potential candidate biomarkers in the discovery set were generated using MetaboAnalyst 5.0 software. A two-tailed *P* < 0.05 was considered statistically significant.

Patient demographic data were examined using SPSS software version 25.0 (SPSS Inc., Chicago, IL). Differences in gender were determined by using Fisher’s exact test. One-way analysis of variance (ANOVA) and Mann-Whitney *U* test were performed for multiple comparisons. Receiver operator characteristic (ROC) analyses were used to predict the diagnostic efficiency of the different metabolites. A *P*-value < 0.05 was considered a criterion for statistically significant differences.

## Result

### Clinical characteristics of participants

Sixty-five patients met the inclusion criteria and were divided into three different groups. No significant differences in age or sex were observed among the three groups (Table 1). The total bile acid level in patients with *S. japonicum* infection was higher than that in healthy individuals. Furthermore, patients with advanced *S. japonicum* infection exhibited even higher levels of bile acids compared to those with chronic infection (Table 1). Compared to the healthy control group, patients with *S. japonicum* infection showed an increased level of blood glucose, total cholesterol and low-density lipoprotein (Table 1). Nevertheless, there were no significant differences between ASJ and CSJ. The clinical characteristics of the participants are shown in Table 1.

### Metabolomic analysis of *S. japonicum* infection sera

**Fig. 1:**
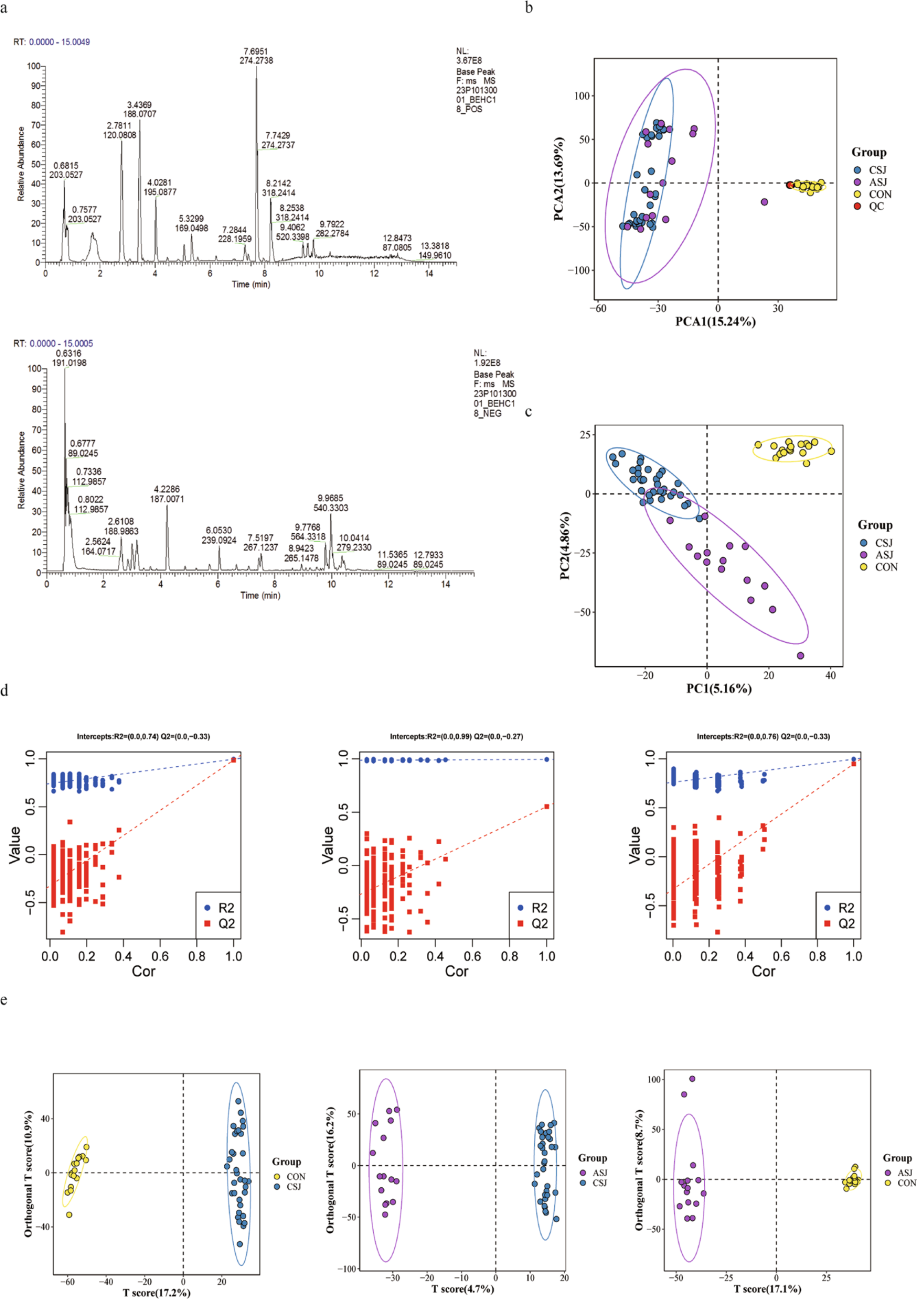
Metabolomic analysis of *Schistosoma japonicum* infection serum. **a** Overlapping of the BPCs (base peak chromatograms) of all QC samples. The results of good chromatograms overlap; little fluctuation of retention time and peak response intensity showed that the instrument is in good condition and the signal is stable throughout the whole process of sample detection and analysis. **b** PCA score plot between the healthy control and *S. japonicum* infection groups. **c** PLS-DA score plot between the healthy control and *S. japonicum* infection groups. **d** To judge the quality of the model without fitting risk, 200 response permutation tests (RPT) were performed on the OPLS-DA model. In this figure, the two points in the upper right corner represent R2 and Q2 of the actual model. The dot on the left represents the displacement test result. Q2 obtained by displacement test is less than Q2 of the model. A red dotted line inclines upward; this indicates that the model is good and has not been fitted. **e** OPLS-DA score plot between the healthy control and *S. japonicum* infection groups. CON: healthy control; CSJ: chronic *S. japonicum* infection; ASJ: advanced *S. japonicum* infection

The overlaid base peak intensity chromatograms of the QC samples in positive and negative ion modes verified the reliability and stability of the system (Fig. 1a). PCA indicates variability among CON, CSJ and ASJ (Fig. 1b). The observed differences among the samples, as depicted in the figure, can be attributed to variations in metabolite levels. Then, PLS-DA was applied to these three groups of biological samples to establish the relationship model between metabolite expression and sample class, which can realize the modeling and prediction of sample class (Fig. 1c). OPLS-DA was performed on the collected data to further identify variations in the three groups. Two hundred iterations of the response permutation test showed negative Q^2^ intercept values (Fig. 1d), indicating that the OPLS-DA model was not overfit. OPLS-DA score plots showed great separation, confirming significant differences among the three groups (Fig. 1e).

### Differential metabolites in different stages of *S. japonicum* infection

**Fig. 2:**
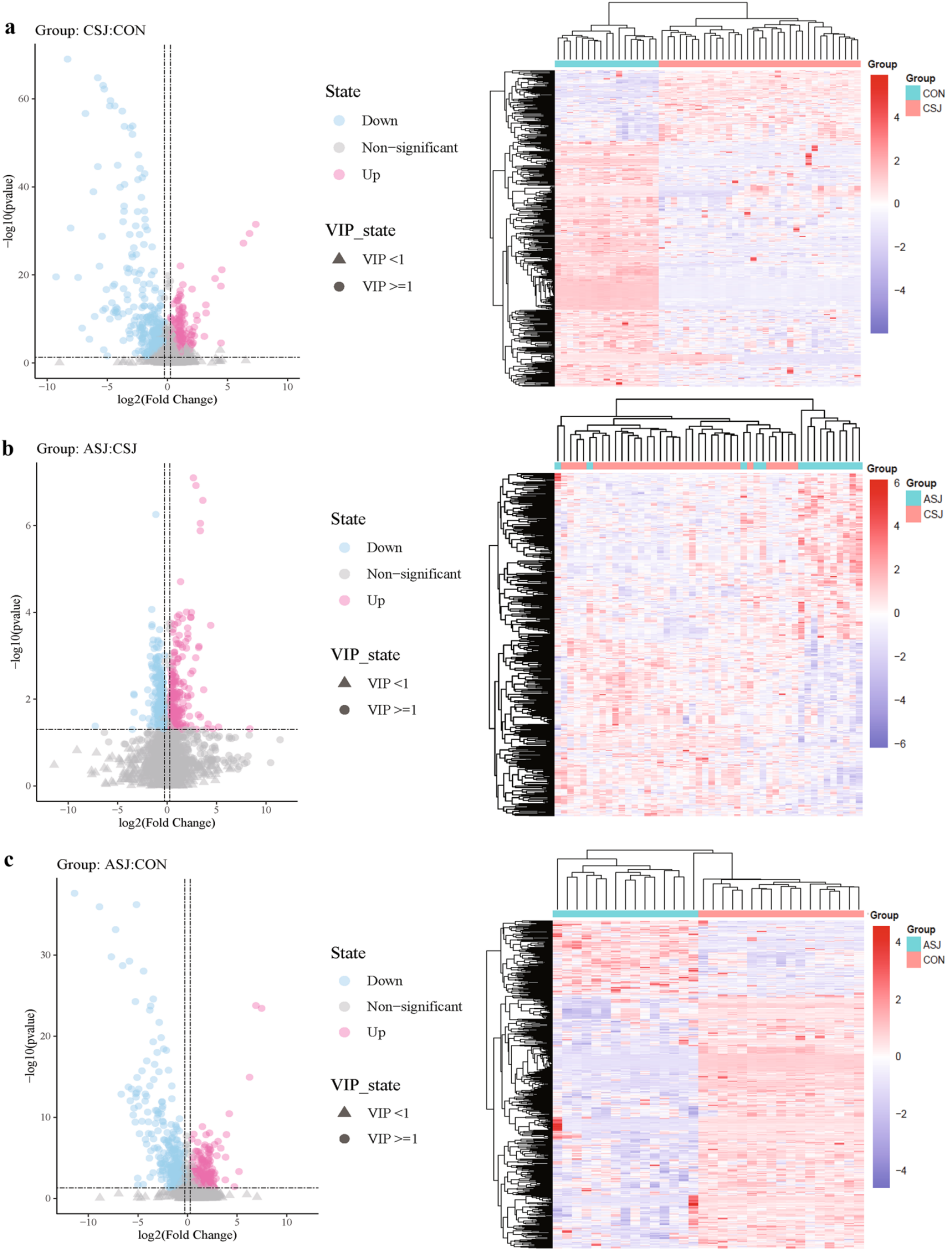
Differential metabolites in different stages of *Schistosoma japonicum* infection. In the volcano, the abscissa is fold change converted by log2 and the ordinate is *P*-value converted by -log10. Blue is the downregulated significant difference metabolite. Red is the upregulated significant difference metabolite. The circle shape is the metabolite with VIP ≥ 1. The triangle is the metabolite with VIP < 1, and the non-significant metabolite is gray. In the heatmap, each row represents a differential metabolite, and each column represents a sample. The color represents the expression level, and blue to red corresponds to the expression level from low to high. **a** Differential metabolites between patients with chronic *S. japonicum* infection and healthy controls; **b** differential metabolites between patients with chronic *S. japonicum* infection and advanced *S. japonicum* infection; **c** differential metabolites between patients with advanced *S. japonicum* infection and healthy controls. CON: healthy control; CSJ: chronic *S. japonicum* infection; ASJ: advanced *S. japonicum* infection

Herein, we conducted a differential analysis of high-quality metabolites to gain quantitative insights into their relative levels among samples from the different groups. Differential metabolites were identified based on specific criteria: FC ≥ 1.2 or FC ≤ 0.83, *P*-value < 0.05, and VIP ≥ 1.0. Comparing samples of CSJ with healthy controls, we found 185 upregulated metabolites and 297 downregulated metabolites. The significant metabolites mainly belonged to categories such as benzene and derivatives, organoheterocyclic compounds, glycerophospholipids, bile acids, alcohols and derivatives, and fatty acyls (Fig. 2a and S1). Furthermore, comparing the ASJ to the CSJ, we identified 199 upregulated metabolites and 207 downregulated metabolites in ASJ. These differentially abundant metabolites were categorized as steroids and derivatives, glycerophospholipids, amino acids, peptides and analogs, organoheterocyclic compounds, organic acids and fatty acyls (Fig. 2b and S2). Comparing the ASJ and healthy control groups, we found 187 upregulated metabolites and 383 downregulated metabolites in ASJ. These metabolites were classified in categories such as benzene and derivatives, steroids and derivatives, organoheterocyclic compounds, fatty acyls, glycerophospholipids, terpenoids and carbohydrates (Fig. 2c and S3).

### Functional enrichment analysis of the differentially expressed metabolites

**Fig. 3:**
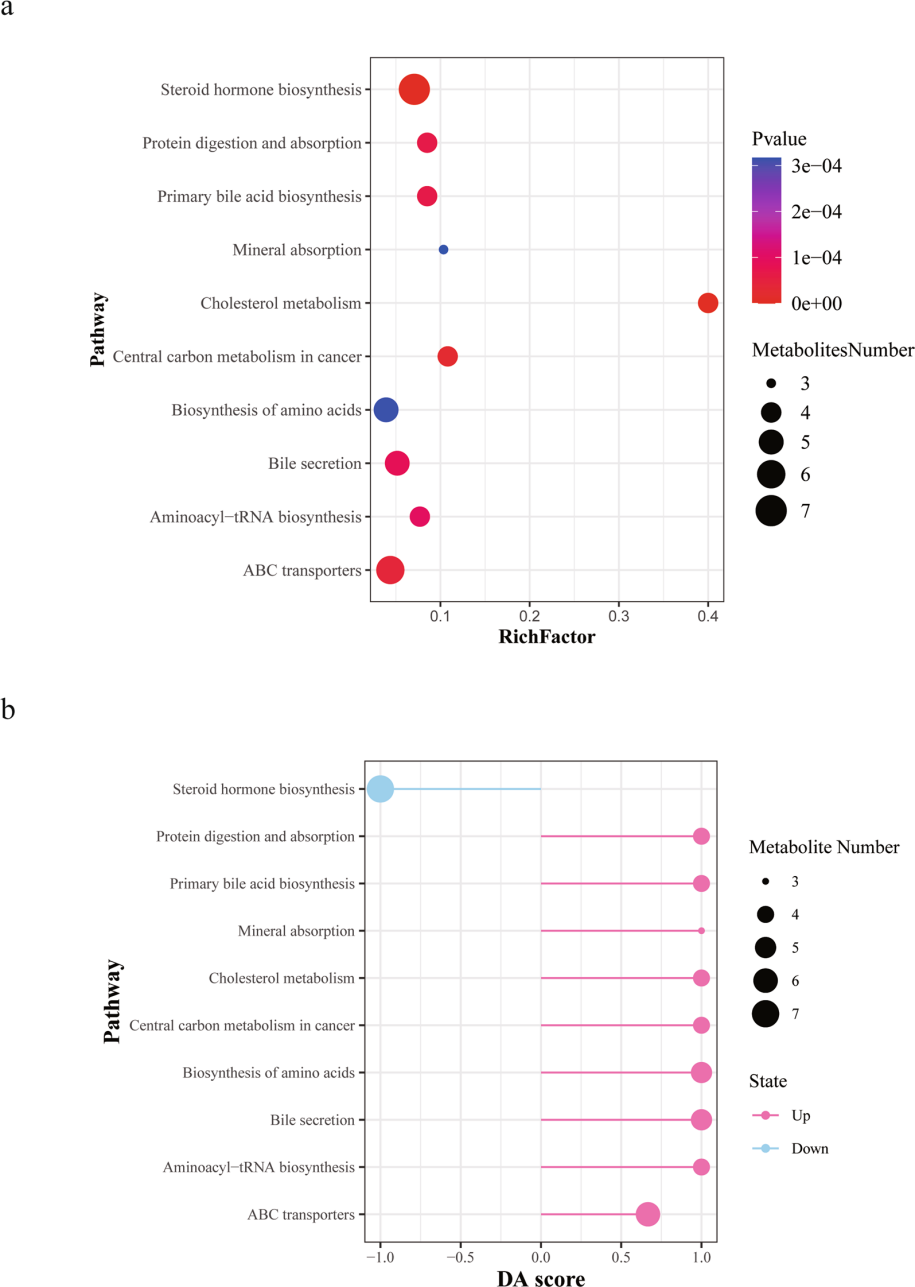
Functional enrichment analysis of the differentially expressed metabolites. **a** X-axis rich factor is the number of differential metabolites annotated in this pathway divided by all identified metabolites annotated in this pathway. The higher the value, the higher the ratio of differential metabolites annotated in this pathway is. The dot size represents the number of differential metabolites annotated in this pathway. **b** Y-axis in the figure represents the name of the metabolite pathway, and X-axis coordinate represents the DA score. DA score is the overall total change of all metabolites in a metabolite pathway. A score of 1 indicates that the expression trend of all annotated differential metabolites in the pathway is upregulated, and a score of negative 1 indicates that the expression trend of all annotated differential metabolites in the pathway is downregulated. The length of the line segment represents the absolute value of the DA score, the size of the dot at the endpoint of the line segment represents the number of metabolites in the pathway, and the larger the dot, the greater the number of metabolites

Using KEGG, we expressed metabolites in different stages of *S. japonicum* infection and found that 10 pathways are significantly enriched in CSJ and ASJ, including steroid hormone biosynthesis, cholesterol metabolism, bile secretion and primary bile acid biosynthesis (Table 2 and Fig. 3a). DA score is a pathway-based metabolite change analysis method, which can help to understand the overall changes in metabolite pathways [21]. Compared with the CSJ, DA score analysis indicated that the steroid hormone biosynthesis pathway was significantly downregulated in ASJ; pathways such as cholesterol metabolism, bile secretion, ATP-binding cassette (ABC) transporters, amino acid biosynthesis and primary bile acid biosynthesis were significantly upregulated (Fig. 3b).

**Table 2:** KEGG pathway analysis shows the metabolism pathway between ASJ and CSJ

Pathway	Count	Count all	*P* value	Pathway ID	KEGG names	logPvalue
Cholesterol metabolism	4	10	8.66E-08	map04979	Glycocholic acid;taurochenodeoxycholic acid;glycochenodeoxycholate;taurocholic acid	16.26188808
Steroid hormone biosynthesis	7	99	3.65E-07	map00140	Desoxycortone;tetrahydrocortisone;androsterone glucuronide;etiocholanolone;androsterone;5a-pregnan-3,20-dione;dehydroepiandrosterone (DHEA)	14.82274375
Central carbon metabolism in cancer	4	37	2.48E-05	map05230	Citrate;l-asparagine;l-histidine;l-methionine	10.60586399
ABC transporters	6	137	4.09E-05	map02010	Spermidine;l-threonine;d-xylose;l-histidine;4-hydroxyproline;inosine	10.10558244
Primary bile acid biosynthesis	4	47	6.45888E-05	map00120	Glycocholic acid;taurochenodeoxycholic acid;glycochenodeoxycholate;taurocholic acid	9.647469227
Protein digestion and absorption	4	47	6.45888E-05	map04974	l-asparagine;l-threonine;l-histidine;l-methionine	9.647469227
Bile secretion	5	97	8.55849E-05	map04976	Spermidine;glycocholic acid;taurochenodeoxycholic acid;glycochenodeoxycholate;taurocholic acid	9.366002276
Aminoacyl-tRNA biosynthesis	4	52	9.63E-05	map00970	l-asparagine;l-threonine;l-histidine;l-methionine	9.247733772
Biosynthesis of amino acids	5	128	3.14E-04	map01230	Citrate;l-asparagine;l-threonine;l-histidine;l-methionine	8.065397131
Mineral absorption	3	29	3.17E-04	map04978	l-asparagine;l-threonine;l-methionine	8.056306622

### Metabolites associated with the progression of *S. japonicum* infection

**Fig. 4:**
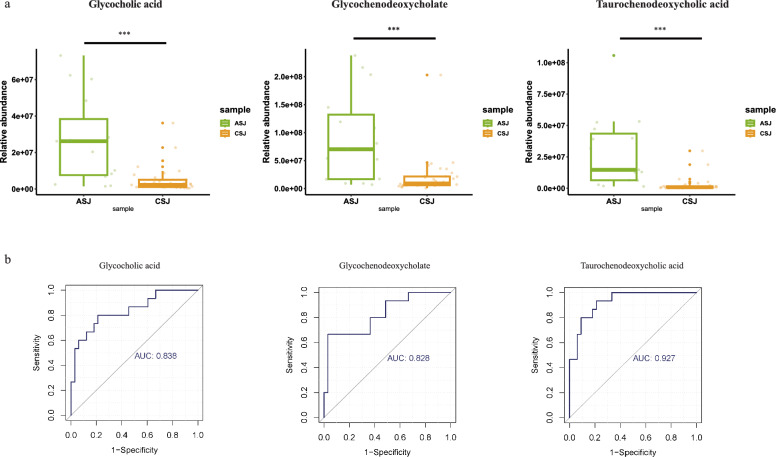
Metabolites associated with the progression of *Schistosoma japonicum* infection. **a** Focus key metabolite change associated with *S. japonicum* infection progression. Mann-Whitney U test was used to compare two independent samples. ***P < 0.001; **b** ROC analysis on differential metabolites. X axis represents 1-specificity; y-axis represents sensitivity. The area under the curve is the AUC value. A higher AUC indicates the metabolite is a better biomarker. *CSJ* chronic *S. japonicum* infection, *ASJ* advanced *S. japonicum* infection

To identify metabolites that have potential to assist diagnosis of different stages of *S. japonicum* infection, we performed further analysis on the different expressed metabolites. A Venn diagram analysis revealed that there were 133 different metabolites when comparing CON and CSJ samples, 233 when comparing CSJ and ASJ samples and 174 when comparing ASJ and CON samples (Figure S4 and Table S1–3). These findings suggest that there are relatively fewer different metabolites associated with progression to advanced schistosomiasis. Specifically, we noticed that glycocholic acid (GCA), glycochenodeoxycholate (GCDCA) and taurochenodeoxycholic acid (TCDCA) exhibited a gradual rise in concentration as the disease progressed (Fig. 4a). To evaluate the predictive ability of potential biomarkers for advanced *S. japonicum* infection, we performed ROC analysis on different expressed metabolites. Our study identified three metabolites with area under the curve (AUC) > 0.8, including GCA, GCDCA and TCDCA, concentrated in cholesterol metabolism, biliary secretion and primary bile acid biosynthesis (Table 2). Thus, ROC analysis indicated that GCA, GCDCA and TCDCA seemed to have potential to act as candidate biomarkers for diagnosis of advanced *S. japonicum* infection (Fig. 4b).

## Discussion

Schistosomiasis remains a public health problem in the world. Although there have been advances in the diagnosis of schistosomiasis, the methods used in the clinic are mainly conventional diagnostic technologies, characterized by low sensitivity and specificity. The main goal of controlling schistosomiasis is not only the development of novel diagnostic tools but also the implementation of the WASH measurements among mass drug administrations, which are stated in WHO Roadmap 2030 [23]. Moreover, the mechanism of the progression of *S. japonicum* infection from chronic to advanced stage is still not well defined. The metabolome has been reported to be important in various disease diagnoses and prognoses, and few studies have also addressed the metabolite status of clinical *S. japonicum* infection patients [13], but the role of metabolite profiles in the progression of *S. japonicum* infection in the clinic is not clear. In this study, we used UPLC-MS-based method to detect the serum metabolite profiles in patients with *S. japonicum* infection. Our results demonstrated that patients in different stages of *S. japonicum* infection had different metabolite signatures. In addition, our study revealed that steroid hormone biosynthesis, cholesterol metabolism and amino acid metabolism possibly become involved in the progression from chronic to advanced *S. japonicum* infection. Furthermore, ROC analysis indicated that GCA, GCDCA and TCDCA have potential to be novel metabolite biomarkers to differentiate patients with chronic from advanced *S. japonicum* infection.

Fluctuations in steroid hormone levels can greatly affect the diagnosis and prognosis of liver disease [24]*. Schistosoma japonicum* infection leads to reprogramming of glucose and lipid metabolism in the mouse colon [25]. The major differentially expressed metabolite molecules observed in this study were lipids, consistent with previous studies [26, 27]. *Schistosoma japonicum* expresses steroid hormone receptors on the surface, displaying the ability to process steroid hormones to anabolic derivatives [28]. The genome of *Schistosoma* encodes putative enzymes that convert progesterone and pregnenolone to estriol, estrone, androsterone and testosterone [28]. Therefore, *Schistosoma* may exploit this pathway during the developmental stages of their parasitic hosts. Our results indicated a significant decrease in the steroid hormone biosynthesis pathway in patients with advanced *S. japonicum* infection, particularly in the levels of sex hormones, such as androsterone, etiocholanolone and its derivatives, as well as dehydroepiandrosterone. Research on the direct effects of androsterone levels on liver disease was limited. However, studies have established a correlation between the decline of sexual hormone levels and late stages of fibrosis in non-alcoholic fatty liver disease (NAFLD), including androsterone sulfate, epiandrosterone sulfate and dehydroepiandrosterone sulfate [29]. Epiandrosterone is a natural byproduct of dehydroepiandrosterone (DHEA) that is formed through 5α-reductase enzyme. Typically, DHEA is present in the form of dehydroepiandrosterone sulfate (DHEA-S). A previous study found that patients with advanced nonalcoholic fatty liver disease (NASH) had lower levels of DHEA-S [30]. Earlier studies have suggested that administering DHEA to experimental animals can alleviate liver damage by hindering the activity of various inflammatory mediators, including tumor necrosis factor-α and macrophage mitogen inhibitory factor, and by averting the elevation of serum ALT levels [31]. According to the aforementioned research, the decrease in steroid hormone synthesis may diminish the protective effects of these hormones on the liver, consequently aggravating the progression of liver damage related to *S. japonicum* infection.

The clinical data showed that advanced *S. japonicum* infection patients displayed notably elevated serum bile acid levels. Meanwhile, the metabolomics investigation further underscored the substantial upregulation of pathways associated with cholesterol metabolism, primary bile acid synthesis and bile secretion in these advanced *S. japonicum* infection patients. However, it remains unclear whether the increased serum bile acids were the result of liver damage induced by *S. japonicum* eggs or the causes of progressed liver disease; thus, further study is needed. Earlier studies have already indicated that disruptions in cholesterol metabolism can lead directly to liver damage and potentially trigger the development of metabolite liver diseases [32]. Another consequence of heightened cholesterol metabolism is the escalation of bile acid synthesis [33]. Conditions like cholestasis and metabolite disorders have been linked to the accumulation of bile acids at elevated concentrations, which in turn could exert cytotoxic and pro-inflammatory effects on liver tissues [33]. Therefore, the interplay of cholesterol metabolism, bile acid synthesis and bile secretion pathways could indicate crucial mechanisms underlying the progression from chronic to advanced stages of *S. japonicum* infection-related liver disease. Of note, recent animal studies demonstrated that *S. japonicum* infection of mice lacking Farnesoid X receptor (FXR) in hepatocytes increased bile acid toxicity and inhibited autophagy in host hepatocytes, thus possibly accelerating the progression of *S. japonicum* infection by promoting hepatocyte injury [34]. Therefore, it is feasible to treat advanced *S. japonicum* infection by inhibiting cholesterol metabolism, bile acid synthesis and bile secretion-related pathways; however, it requires further animal studies and needs to be validated in patients.

In the pathways that were upregulated, we observed an unexpected surge in the expression of ABC transporters among patients with advanced *S. japonicum* infection. Previous study has indicated that ABC transporters play a pivotal role in modulating the sensitivity to drugs like praziquantel (PZQ) in *Schistosoma* infection as well as other parasitic worms [35]. Thus, ABC transporter inhibitors seemed to have potential benefits for the treatment of *S. japonicum* infection, although this warrants further study. In addition, our analysis revealed increased expression of specific amino acids including l-asparagine, l-threonine, l-histidine and l-methionine in ASJ (Table S2). Similar disruptions in amino acid metabolism have been identified not only in hosts infected with *S. japonicum* infection but also in patients suffering from a range of metabolite liver diseases, along with their corresponding disease models [36–42]. This suggests that targeting these amino acid metabolite abnormalities seemed have potential to provide some evidence for developing novel therapeutic strategies to mitigate liver damage in advanced *S. japonicum* infection patients.

The cytotoxic effect of bile acids is dependent on the composition of the bile acid pool, particularly hydrophobic bile acids exhibiting greater toxicity [43]. Bile acids currently hold significant promise as predictive markers in the progression trajectory of numerous liver diseases [44]. For instance, alterations in bile acid composition are strongly linked with conditions like cirrhosis [45]. Patients with cirrhosis have a lower proportion of bile acids compared to other patients, while the proportion of primary bile acids is higher in patients with advanced liver cirrhosis [46–49]. Recent research on serum metabolite groups has identified 16 bile acids, such as GCA, GCDCA and TCDCA, which are increased in patients with severe hepatitis B (Child-Pugh score) [50]. Besides, a recent study found that the bile acid levels of NASH patients were higher than in healthy controls [50, 51]. Moreover, in patients with cirrhosis, the levels of primary conjugated bile acids such as GCA, GCDCA and TCDCA were significantly elevated compared with non-cirrhotic patients [51]. These results suggested that an increase in primary conjugated bile acids is a characteristic feature of cirrhosis, irrespective of the presence or absence of hepatocellular carcinoma (HCC) [51]. In addition, the study also discovered the expression of GCA, GCDCA and TCDCA induced *TGFβ1* in vitro hepatic stellate cells (HSCs), which indicated that primary conjugated bile acid is related to HSC activation and fibrosis in liver cirrhosis [51]. Consistent with previous studies, our study found that the robust area AUC values of primary conjugated bile acids like GCA, GCDCA and TCDCA were > 0.8. This suggests that variations in these bile acid levels hold promising diagnostic potential for assessing the extent of liver damage in both advanced and chronic *S. japonicum* infection. However, to pinpoint the most pivotal differential metabolites in the progression from chronic to advanced *S. japonicum* infection, further studies are required and need to be validated by large sample size clinical investigation. This will allow for a more in-depth understanding of the metabolite mechanisms underlying the transition from chronic to advanced *S. japonicum* infection.

## Conclusions

UPLC-MS technology represents a novel approach for detecting changes in the serum metabolite profile of individuals with chronic or advanced *S. japonicum* infection. Our study revealed that patients in different stages of *S. japonicum* infection had different metabolome signatures. Furthermore, we found that the steroid hormone biosynthesis, cholesterol metabolism and partial amino acid metabolism correlated with the progression from chronic to advanced *S. japonicum* infection. Notably, our results indicated that GCA, GCDCA and TCDCA seemed to have potential to be novel metabolite biomarkers to differentiate patients with chronic *S. japonicum* infection from those with advanced *S. japonicum* infection, and this research also shed light on the mechanism of the progression from chronic to advanced *S. japonicum* infection in terms of metabolism. However, further studies in larger groups of patients with *S. japonicum* infection are needed to validate the potential role of the metabolites in the progression of *S. japonicum* infection. Overall, our study demonstrates the potential utility of UPLC-MS technology for identifying biomarkers associated with *S. japonicum* infection progression, which could have important implications for clinical management and the development of more effective treatments.

### Supplementary Information

Supplementary Material 1: Fig. S1. Final class of differential metabolites between CON and CSJ groups.

Supplementary Material 2: Fig. S2. Final class of differential metabolites between CSJ and ASJ groups.

Supplementary Material 3: Fig. S3. Final class of differential metabolites between CON and ASJ groups.

Supplementary Material 4: Fig. S4. Venn diagram results among the three groups, CON vs. CSJ, CSJ vs. ASJ and ASJ vs. CON.

Supplementary Material 5: Table S1.Molecular information of differential metabolites between healthy_control and CSJ groups_ESM.

Supplementary Material 6: Table S2.Molecular information of differential metabolites between CSJ and ASJ groups_ESM.

Supplementary Material 7: Table S3.Molecular information of differential metabolites between healthy_control and ASJ groups_ESM.

## Data Availability

The untargeted metabolomic data used in this publication are available from the corresponding author upon reasonable request.

## Abbreviations

*S. japonicum*: *Schistosoma japonicum*
UPLC-MS: Ultra-performance liquid chromatography-mass spectrometry
ROC: Receiver operator characteristic
GCA: Glycocholic acid
GCDCA: Glycochenodeoxycholate
TCDCA: Taurochenodeoxycholic acid
KK: Kato-Katz
PCA: Principal component analysis
PLS-DA: Partial least squares discriminant analysis
OPLS-DA: Orthogonal partial least squares discriminant analysis
VIP: Variable importance for projected
FC: Fold change
KEGG: Kyoto Encyclopedia of Genes and Genomes
ABC: ATP-binding cassette
AUC: Area under the curve
