# Supplementary figures and images for: Metabolomics analysis of patients with Schistosoma japonicum infection based on UPLC-MS method
Source: Parasit Vectors. 2024 Aug 20;17:350. doi: 10.1186/s13071-024-06429-9 (PMC11334362; doi:10.1186/s13071-024-06429-9)

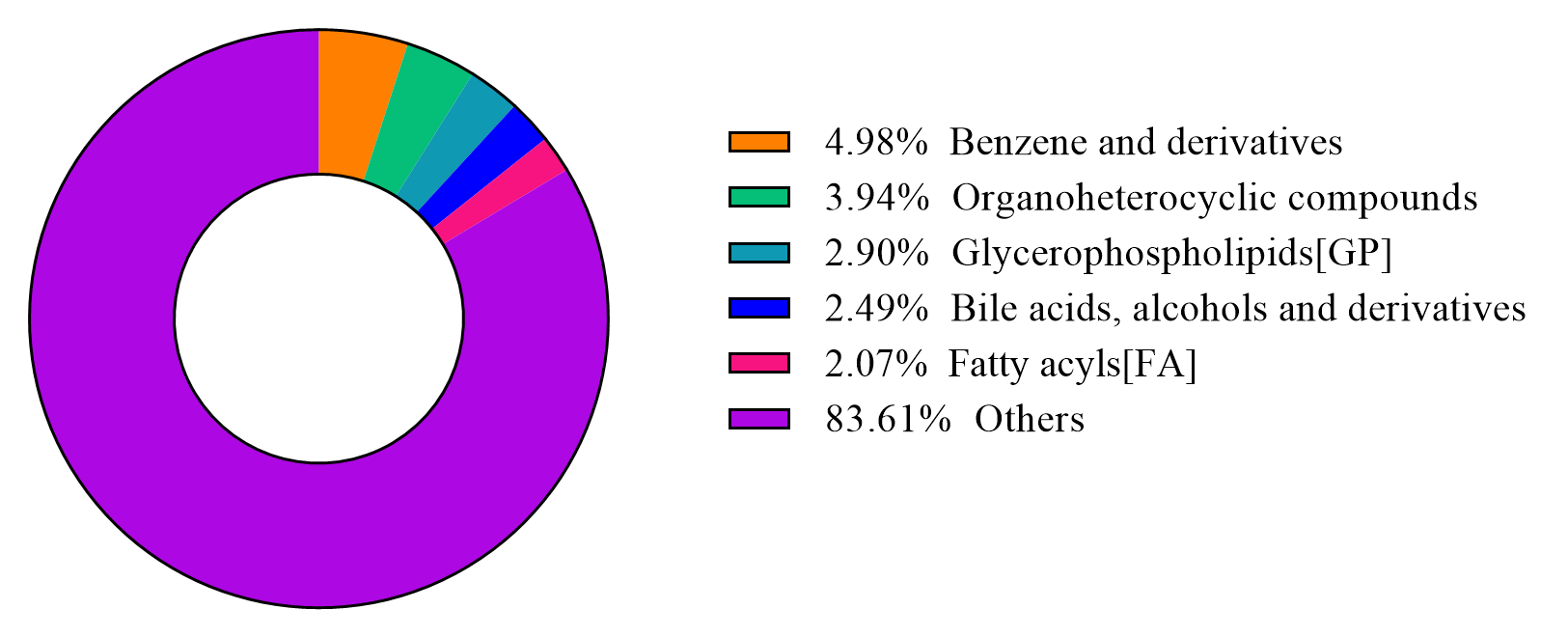

Supplement: Supplementary file 1 — Supplementary Material 1: Fig. S1. Final class of differential metabolites between CON and CSJ groups. [file 13071_2024_6429_MOESM1_ESM.tif]

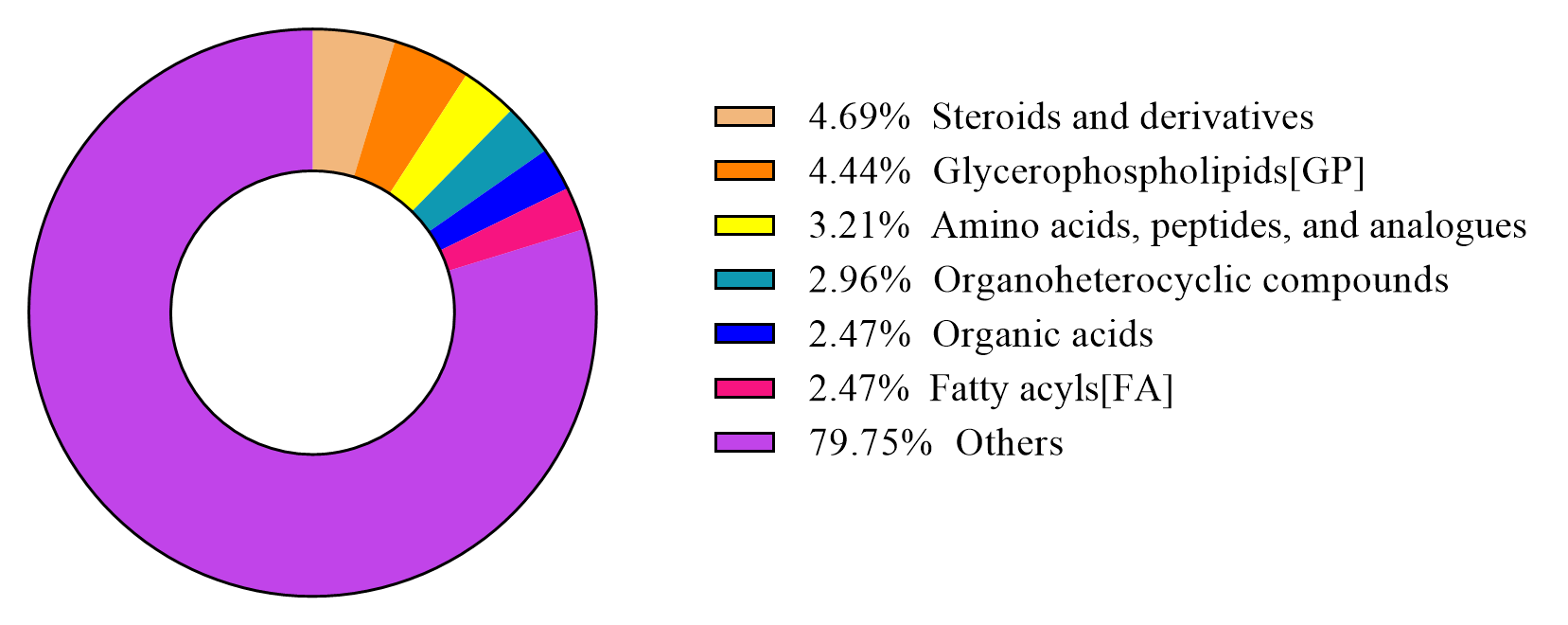

Supplement: Supplementary file 2 — Supplementary Material 2: Fig. S2. Final class of differential metabolites between CSJ and ASJ groups. [file 13071_2024_6429_MOESM2_ESM.tif]

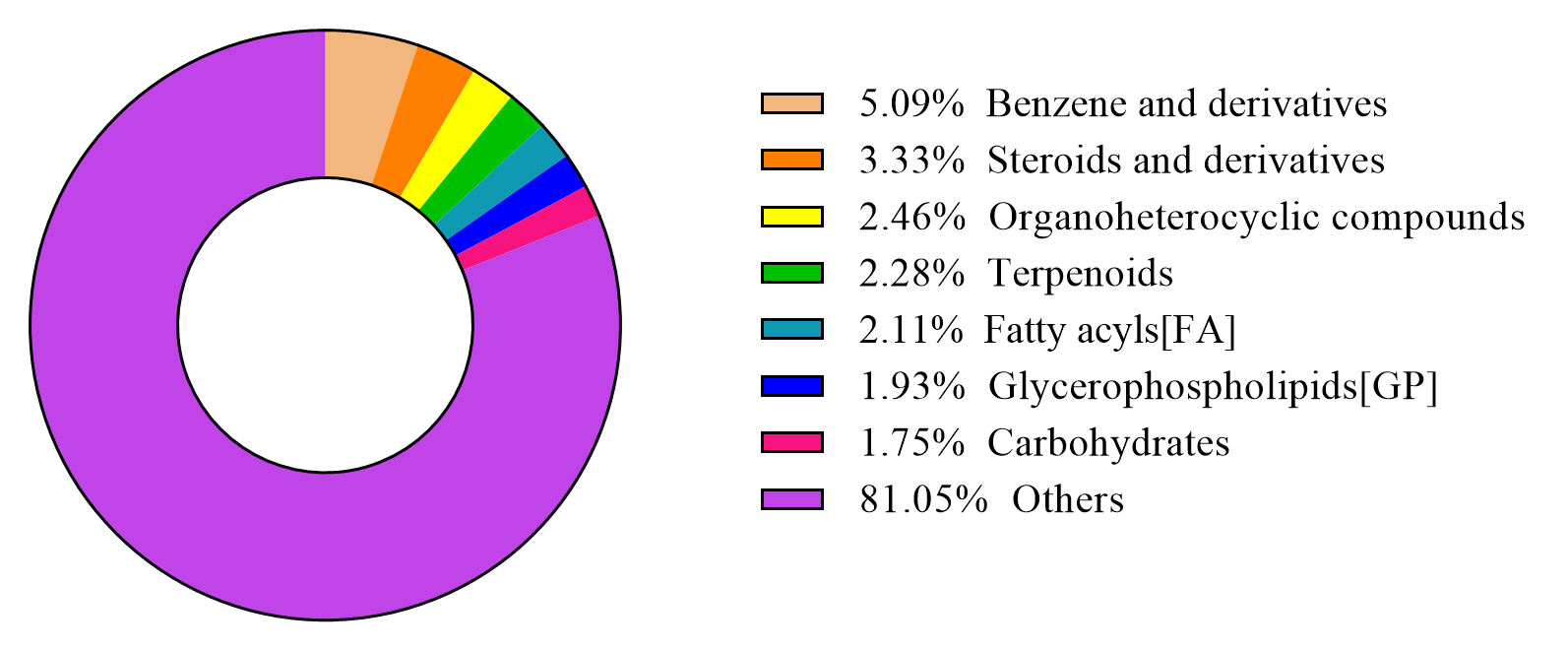

Supplement: Supplementary file 3 — Supplementary Material 3: Fig. S3. Final class of differential metabolites between CON and ASJ groups. [file 13071_2024_6429_MOESM3_ESM.tif]

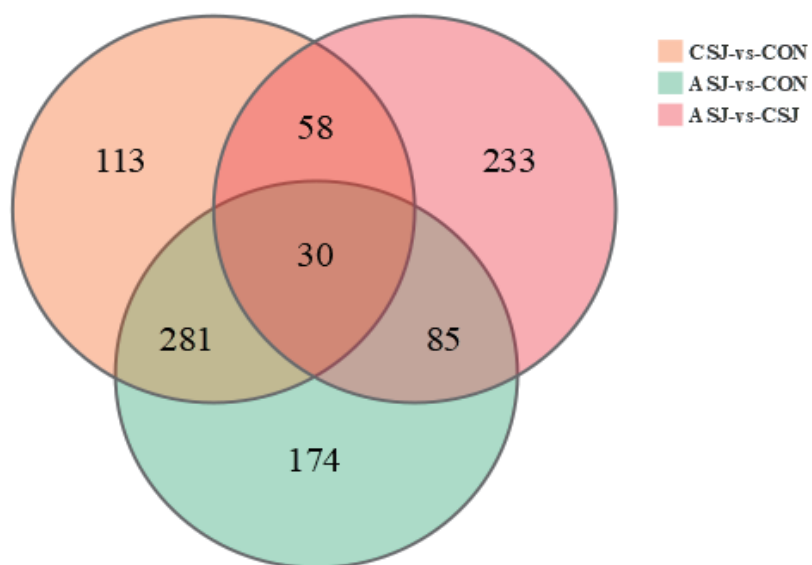

Supplement: Supplementary file 4 — Supplementary Material 4: Fig. S4. Venn diagram results among the three groups, CON vs. CSJ, CSJ vs. ASJ and ASJ vs. CON. [file 13071_2024_6429_MOESM4_ESM.pdf]
